# Deciphering a Changing Match Environment in Emergency Medicine and Identifying Residency Program Needs

**DOI:** 10.5811/westjem.2022.11.58060

**Published:** 2023-01-16

**Authors:** Tiffany Murano, Moshe Weizberg, Bo Burns, Laura R. Hopson

**Affiliations:** * Columbia University Irving Medical Center, Department of Emergency Medicine, New York, New York; † Maimonides Medical Center, Department of Emergency Medicine, Brooklyn, New York; ‡ University of Oklahoma School of Community Medicine, Department of Emergency Medicine, Tulsa, Oklahoma; § University of Michigan Medical School, Department of Emergency Medicine, Ann Arbor, Michigan

## Abstract

**Introduction:**

The Match in emergency medicine (EM) is historically competitive for applicants; however, the 2022 residency Match had a large number of unfilled positions. We sought to characterize the impact of and response to the Match on programs and determine programs’ needs for successful recruitment strategies.

**Methods:**

We conducted a web-based survey of EM residency program leadership during March–April 2022. Program characteristics were generated from publicly available data, and descriptive statistics were generated. We analyzed free-text responses thematically.

**Results:**

There were 133/277 (48%) categorical EM residency programs that responded. Of those, 53.8% (70/130) reported a negative impression of their Match results; 17.7% (23/130) positive; and the remainder neutral (28.5%; 37/130). Three- and four-year programs did not differ in their risk of unfilled status. Hybrid programs had a higher likelihood of going unfilled (odds ratio [OR] 4.52, confidence interval [CI] 1.7–12.04) vs community (OR 1.62, CI 0.68–3.86) or university programs (0.16, 0.0–0.49). Unfilled programs were geographically concentrated. The quality of applicants was perceived the same as previous years and did not differ between filled and unfilled programs. Respondents worried the expansion of EM residency positions and perceptions of the EM job market were major factors influencing the Match. They expressed interest in introducing changes to the interview process, including caps on applications and interviews, as well as a need for more structural support for programs and the specialty.

**Conclusion:**

This survey identifies impacts of the changed match environment on a broad range of programs and identifies specific needs. Future work should be directed toward a deeper understanding of the factors contributing to changes in the specialty and the development of evidence-based interventions.

## INTRODUCTION

The National Resident Matching Program (NRMP) (the Match) in emergency medicine (EM) is historically considered competitive for applicants. By 2021, EM grew to comprise up to 8.1% of positions in the Match with nearly 100% program match rates.^1^ However, the 2022 Match resulted in an unusually large number of unfilled programs and positions.^2^

The application and matching environment for EM has evolved over the past decade. The number of Accreditation Council for Graduate Medical Education (ACGME)-accredited EM training programs and associated training slots increased dramatically since 2016. Of the 95 new programs accredited by the ACGME since 2016, 41 were previously accredited by the American Osteopathic Association (AOA) and received ACGME-accreditation through the single accreditation system moving their positions and applicants into the Match.^3^ In 2021, there were 273 residency programs, with 2,826 of the 2,840 EM positions (99.5%) filled in the Match, and only nine programs (3.3%) unfilled.^1^ In 2022, of the 2,921 offered positions in 277 programs, there were 219 unfilled positions (7.5%), with 69 programs (24.9%) not filling in the Match.^2^ This unprecedented number of unfilled positions calls for reflection on the evolving landscape of EM and residency training.

We sought to understand the needs and perceptions of the specialty’s residency training programs. The Council of Residency Directors in EM (CORD) conducted a survey to characterize the impact and response of programs to the 2022 EM Match. We hypothesized that the impact of the 2022 Match extended beyond the unfilled programs and that we could identify common needs to tailor future support interventions for all our programs.

## METHODS

We conducted a survey of residency training program leadership (ie, program directors [PD], assistant/associate [APD], program coordinators, vice chairs of education) using a web-based survey distributed by Qualtrics (Seattle, WA) during the CORD Academic Assembly in March 2022 in San Diego, CA. Participation in the survey was solicited using QR codes during conference presentations, and the survey was also sent on the organization’s PD listserv during and after the conference to elicit responses from individuals who did not attend the conference.

The survey included program demographic information and respondent’s role. Participants were also asked to indicate whether their program filled in the Match and whether it filled at, below, or above the expected level on their rank list. They were also asked to rank factors they believed may have contributed to the outcome. We created the survey based on knowledge of the literature and current conversations within the specialty. We all have significant expertise in residency administration with more than 50 cumulative years in residency leadership and ongoing involvement with EM education. The survey was pilot-tested with an expanded group of expert volunteers consisting of four current and former PDs, all of whom have experience with survey design. We refined the survey on their feedback prior to distribution. The survey is included as an Appendix. The study was reviewed by the University of Michigan Institutional Review Board and given exempt status.

We analyzed the data using Microsoft Excel 365 (Microsoft Corporation, Redmond, WA) to calculate descriptive statistics and an online calculator for odds ratios (OR) and confidence intervals (CI).^4^

**Population Health Research Capsule**

What do we already know about this issue?

*The 2022 residency Match in emergency medicine had a large number of unfilled positions. No previous research has been published to explain the sudden change in the Match outcome.*

What was the research question?

*What was the impact of the 2022 Match on EM residency programs? What needs do programs have for the future?*

What was the major finding of the study?

*Perceived worsened Match results were mainly attributed to increased slots and the future job market. Application and interview processes were a major concern.*

How does this improve population health?

*These findings will help the specialty in developing program-level resources that will address future needs.*

To avoid over-weighting perspectives from a single program, data were sorted to select a single response per program. We used the following order of consideration to select responses when more than one was available per program: residency PD; residency coordinator; vice (or associate) chair or chair; APD; residency core faculty member; general faculty member. No questions were required in the survey. We used the American Medical Association’s publicly available portions of their Fellowship and Residency Electronic Interactive Database (FREIDA) of residency programs for self-reported demographic descriptions, and the Emergency Medicine Residents Association Match for training format.^5^,^6^ Free-text responses were coded by consensus between two authors (MW, TM) using descriptive codes, and any disagreements were resolved by the other two authors (BB, LRH). The codes were then grouped into broader themes by the entire author group.

## RESULTS

Of an initial 169 responses, 133 represented unique programs for a 48% (133/277) response rate of EM residency programs. Unique respondents for programs included 103 PDs (77.4%); 18 APDs (13.5%); four chairs including vice and associate (3.0%); four coordinators (3.0%); three clerkship leadership (2.3%); and one other (<1%). We compared demographics of responding programs with those of EM residency programs as a whole in Table 1 using the publicly available and program-defined characteristics from the AMA FREIDA database.^5^

Of the 69 unfilled programs, 26 responded to this survey (37.6%). Unfilled programs in our study were predominantly concentrated in four geographic divisions: Mid-Atlantic, East North Central, South Atlantic, and West South Central (92.3%, 24/26). The postgraduate year (PGY) 1–3 format programs were not more affected than PGY 1–4 (OR 2.1; CI 0.58–7.6, ns). Programs self-described as community/university-affiliated (hybrid) programs had a much higher odds ratio at 4.52 (CI 1.7–12.4) of going unfilled in our sample than university (OR 0.16, CI 0.05–0.29) or community programs (OR 1.62, CI, 0.68–3.86). Programs overall viewed their match outcome negatively with 70/130 (53.8%) responding that the overall quality of their match was a little or substantially worse than typical. Even when only filled programs were considered, 51/105 (48.6%) reported that their match outcome was worse than in previous years. A minority reported a better than usual perceived match outcome (23/130 (17.7%) all respondents; 23/105 (22.0%) filled programs only). Programs perceived the quality of applicants as similar to previous years. This did not significantly differ between filled and unfilled programs.

In general, programs reported interviewing an average of 14.2 candidates per residency position available (n=123; range 4.4–30.0, SD 4.0). Unfilled programs averaged 13.9 applicants per residency position (range 8.1–23.5, SD 3.2) with filled programs averaging 14.3 (range 4.4–30.0, SD 4.2), which did not reflect a significant difference (*P*=0.7, ns). Neither did filled and unfilled programs differ significantly in the number of candidates they interviewed but elected not to place on their rank lists. Programs largely indicated that they would continue to interview about the same number of applicants in the next year (68/125 responding, 54.4%), although unfilled programs were more likely than filled programs to indicate plans to expand the size of their interview pool (20/23, 87.0% vs 26/102, 25.5%).

Unfilled programs generally reported a positive outcome with the Supplemental Offer and Acceptance Program (SOAP), with 75% (20/25 responding) reporting they were either extremely or somewhat satisfied with the quality of candidates in the SOAP. Programs averaged 3.2 open positions entering into the SOAP (range 1–10) with an average intern class size of 10 (range 6–16). Fifteen of 25 (60%) had more than one open position in the SOAP. Program needs regarding the SOAP largely centered on rapidly learning an unfamiliar process and the need for guidance and support.

Respondents focused on the expansion of EM residency slots in both new (23.9%) and existing (19.4%) programs, as well as student perceptions of the future job market within EM (21.7%), as the major factors they felt influenced the 2022 Match. The unfilled programs focused on perceptions of future job prospects in EM (48.0%) and the virtual interview format (28.0%) as their major concerns.

Programs identified their greatest needs for the future and resources from the CORD organization in free-text responses. These were grouped into themes (Table 2**)**. The majority of the responses indicated a desire for structural changes in the interview process including placing caps on the number of programs that students may apply to as well as the number of interviews they may attend. Thirty-five programs (36.1%, n=97 total programs responding) indicated that the number of interviews per applicant should be capped. There was also a significant interest in an in-person component to interviews (31/97 responding, 32.0%).

## DISCUSSION

We report data on program perceptions and experiences in the 2022 EM Match. Overall, we identified significant program-level concerns associated with the unprecedented number of unfilled residency positions and the need to unexpectedly use the SOAP. Recognizing that the depth of the rank order list (ROL) is not the same as true applicant quality, programs broadly reported that they went deeper into their ROLs than in previous years. This trend affected both filled and unfilled programs, which suggests that the changes in the Match environment itself are responsible rather than individual program factors. We did not identify a clear difference in numbers interviewed between filled and unfilled programs to account for different outcomes in the Match.

One potential risk factor, which is suggested by our data, is the influence of geography on program outcomes. Our dataset, as well as the general NRMP data, show that the unfilled positions in EM were higher in certain geographic areas.^2^ A 2021 NRMP survey of senior medical students entering EM indicated that desired geographic location was the single most important factor when selecting programs for application and third in importance when ranking programs.^7^ For comparison, the reputation of the program was the third-most important factor in selecting programs to apply to and fifth in ranking programs.^7^ In the 2022 Match, there were a significant number of newer programs that went unfilled, with evidence of clustering of unfilled programs in specific geographic areas.^2^ While we cannot make a direct assessment of the exact importance of each factor, this may also suggest that the most recently approved programs and those located in specific geographic areas are at higher risk to go unfilled in the Match. Programs that fit these descriptions may benefit from more strategic interviewing and recruitment strategies.

Challenges with the application and interview processes were a major concern among our respondents. Programs expressed a desire for changes including interest in the implementation of program signaling, interview control (capping applications and interviews), and allowing an in-person component to the interview day (Table 2). Current NRMP survey data suggests that US allopathic students applying to EM sent applications to 40–49 programs; osteopathic applicants 62–64 programs; and other applicants 95–101 programs.^7^ These application numbers contrast with additional NRMP data that EM applicants ranking 10 programs have almost a 95% chance of matching in the specialty.^8^ On the surface, limiting the number of applications that a student may submit has appeal; however, it creates challenges and may inadvertently disadvantage certain subgroups of applicants. Alternative proposals such as specialty-level caps on the number of residency interviews, such as that being explored by ophthalmology, or phased application cycles, may merit further exploration within EM.^9^–^12^ Preference signaling is already in use by other specialties such as otolaryngology, dermatology, and obstetrics/gynecology.^13^,^14^ Emergency medicine is currently piloting this system for the 2022–2023 application cycle.^15^ Programs are now caught between managing large volumes of applications and the understandable fears evolving out of a difficult match year. Any interventions must respect both of those realities and will require careful implementation to minimize the chances of an overcorrection.

There are undoubtedly external factors that may have influenced how students view the specialty including the dramatically tight job market in 2020 that was precipitated by the financial crisis with COVID-19.^16^ The influence of the COVID-19 pandemic and its direct and indirect impacts on EDs are unknown. There is also the widely publicized emergency physician workforce study that predicted there will be a surplus of almost 8,000 emergency physicians by the year 2030.^17^ We clearly observed a concern from our programs that these factors, coupled with how they were interpreted by and messaged to medical students, may have played a large role in the outcome of the Match this past year.^18^ A more recent study questions the conclusion of a physician surplus, identifying that the attrition rate used (3%) was artificially low and led to an overestimation.^17^,^19^

This is somewhat reminiscent of anesthesiology’s challenges in the 1990s in which a major surplus was predicted.^17^ These estimates were later found to be based on inaccurate assumptions, but the adverse publicity surrounding the predicted oversupply of anesthesiologists led to a dramatic decrease in the number and quality of medical students applying to US anesthesiology residency programs, as students were being advised to choose alternative careers.^20^ This resulted in a massive need for anesthesiologists in the next decade.^21^ Certainly, concerns are being raised about the long-term EM workforce and our rate of graduating new emergency physicians, but the magnitude of these challenges is unclear.^22^,^23^ Our respondents clearly identified priorities to educate students regarding these findings and address concerns about the future viability of our specialty. There were suggestions to limit or even shrink the number of residency positions either by preventing current program expansion or limiting the creation of new programs. Given the evolving data around the state of the EM workforce and cautionary lessons from anesthesiology’s overcorrection two decades ago, we would be wise to use caution in the measures that we take in limiting the number of training positions. However, further study and thoughtful design of interventions to continue to develop the quality and scope of training in the specialty should be pursued.

## LIMITATIONS

This was a voluntary survey subject to selection bias focused on organizational involvement with CORD. However, our 48% response rate is composed of a broad geographic and program-format sampling, which provides support for its conclusions. It is important to note that there are response biases in our sample with overrepresentation of filled programs and some geographic regions, which may have biased results. In addition, the issues around a successful Match are tied to program reputation, and responses may be impacted by social desirability bias given the identified nature of the data. While we recognize that disclosure and discussion of information by specific programs may be sensitive, this information is publicly available through NRMP reports.

## CONCLUSION

We present data from a survey of EM residency program leadership in the wake of the 2022 EM residency match, which identifies broad-based effects extending beyond the historic number of unfilled EM residency programs. In addition, the unfilled programs have needs for support including effective use of the SOAP program. Interventions at the specialty level will include a research agenda and development of program-level resources that will address these needs. Future work should be directed toward a deeper understanding of the factors contributing to these changes and the development of evidence-based policy interventions.

## Supplementary Information



## Figures and Tables

**Table 1:** Emergency medicine (EM) program demographics. Column 1 (All EM programs) represents the complete list/description of ACGME-accredited EM programs. Column 2 (All responding programs; n=133) indicates the total number of programs that responded to the survey. Columns 3 and 4 further break down the filled (n=107) and unfilled programs (n=26) of all respondents, respectively.

	All EM programs (n=277)	All responding programs (n=133)	Filled programs responding (n=107)	Unfilled programs responding (n=26)
Region				
New England	12 (4.3%)	9 (6.8%)	9 (8.4%)	0 (0.0%)
Mid-Atlantic	65 (23.5%)	25 (18.8%)	18 (16.8%)	7 (26.9%)
East North Central	58 (20.9%)	23 (17.3%)	16 (15.0%)	7 (26.9%)
West North Central	11 (4.0%)	7 (5.3%)	7 (6.5%)	0 (0.0%)
South Atlantic	53 (19.1%)	29 (21.8%)	24 (22.4%)	5 (19.2%)
East South Central	11 (4.0%)	3 (2.3%)	3 (2.8%)	0 (0.0%)
West South Central	27 (9.7%)	10 (7.5%)	5 (4.7%)	5 (19.2%)
Mountain	11 (4.0%)	8 (6.0%)	7 (6.5%)	1 (3.8%)
Pacific	27 (9.7%)	18 (13.5%)	17 (15.9%)	1 (3.8%)
Territory	2 (0.7%)	1 (0.8%)	1 (0.9%)	0 (0.0%)
Self-identified program type				
University	94 (33.9%)	61 (22.0%)	57 (53.3%)	4 (15.3%)
Community	49 (17.7%)	23 (8.3%)	37 (34.6%)	12 (46.2%)
Community/ university-affiliated (hybrid)	129 (46.6%)	49 (17.7%)	13 (12.1%)	10 (38.5%)
Military	5 (1.8%)	0 (0.0%)	0 (0.0%)	0 (0.0%)
Training format				
PGY 1–3	220 (79.7%)	107 (80.5%)	84 (78.5%)	23 (88.5%)
PGY 1–4	56 (20.3%)	26 (19.5%)	23 (21.5%)	3 (11.5%)

*ACGME.* Accreditation Council for Graduate Medical Education; *PGY*, postgraduate year.

**Table 2:** Thematic summary of free-text responses for program needs. This table represents themes identified in the free-text answers to program needs from CORD after the 2022 EM Match. The number in parentheses indicates the number of respondents who mentioned that element.

**Needs related to the interviews/recruitment process (# responses)** Interest in central control of residency interviews within the specialty Cap on the number of interviews per applicant (35) Cap on the number of residency applications per applicant (10) Control timing of interview offers such as a universal date (3) Preferences for interview format Hybrid (includes some/any in-person component) (17) All In-person (14) All virtual (2) Interest in preference signaling in the match process (20) Explore changes to the Match structure itself (e.g. early match, SOAP process) (5) Promotion/support of programs within the specialty regardless of size, location, and reputation (5) Enforce adherence to standards of conduct in interviews/match (3) Encourage holistic reviews of applications by programs despite limitations in the quality of information (2)
**Needs related to student advising and experience (# responses)** Develop strategies to educate students regarding the future of EM and potential challenges Help build best practices for innovative ways for EM residents to find jobs (12) Address workforce concerns and take steps to address applicant concerns/dispel rumors and misconceptions (10) Standardize advice to applicants regarding number of applications, interviews, and away rotations (14) Develop strategies to promote the specialty to students (7) Advocate for increased exposure to EM during medical school (3) Provide resources for faculty development around the match processes (2)
**Residency training and resources needs (# responses)** Increased transparency and communication to the programs on information relevant to residency operations and the match including data from recent research, organizational purpose and data releases (eg: NRMP, ACGME, ERAS), ACGME initiatives and decisions affecting programs (8) Increased organizational advocacy for faculty support of residency leadership (3)
**Need related to the future of EM (# responses)** Provide guidance for regulations regarding residency programs’ approval, expansions, and residency format. Specific areas for engagement and exploration include: Capping residency spots and expansion of programs (33) Raising accreditation standards for training programs (12) Increasing accountability for maintaining accreditation standards (4) Promulgating recommendations for a single training format (2) Identifying and tracking training outcomes (1) Structural concerns around the practice of EM Desire to expand scope of practice opportunities for emergency physicians (9) Concerns regarding training programs sponsored by contract management groups (5) Engage with study on the EM workforce including incorporation of new information (4) Competition from advanced practice providers (3)

*CORD*, Council of Residency Directors in Emergency Medicine; *EM*, emergency medicine; *NRMP*, National Residency Match Program; *ACGME*, Accreditation Council for Graduate Medical Education; *ERAS*, Electronic Residency Application Service; *SOAP*, Supplemental Officer and Acceptance Program.

## References

[1] National Resident Matching Program (2021). Results and Data: 2021 Main Residency Match®.

[2] National Resident Matching Program (2022). 2022 NRMP Main Residency Match®: Match Rates by Specialty and State National Resident Matching Program.

[3] Accreditation Council for Graduate Medical Education (ACGME)-Public List of Programs that Applied for Accreditation Under the Single Accreditation System by Specialty.

[4] Odds Ratio Calculator.

[5] Fellowship and Residency Electronic Interactive Database (FREIDA™).

[6] Match EMRA https://webapps.emra.org/utils/spa/match#/search/map.

[7] (2021). National Resident Matching Program^®^, Data Release and Research Committee: Results of the 2021 NRMP Applicant Survey by Preferred Specialty and Applicant Type.

[8] (2020). National Resident Matching Program^®^. Charting Outcomes in the Match: Senior Students of U.S. Medical Schools, 2020.

[9] Burk-Rafel J, Standiford TC (2021). A novel ticket system for capping residency interview numbers: reimagining interviews in the COVID-19 era. Acad Med.

[10] Quillen DA, Siatkowski RM, Feldon S (2021). COVID-19 and the ophthalmology match. Ophthal.

[11] Mattson CD, Farina EM (2021). Use application phases instead of interview caps: Help applicants match into programs in which they are genuinely interested. Acad Med.

[12] Dacre M, Hopson LR, Branzetti J (2021). The Summer Match: a qualitative study exploring a two-stage residency match option. AEM Educ Train.

[13] Salehi PP, Azizzadeh B, Lee YH (2019). Preference signaling for competitive residency programs in the NRMP. J Grad Med Educ.

[14] Pletcher SD, Chang CWD, Thorne MC (2022). The otolaryngology residency program preference signaling experience. Acad Med.

[15] Pelletier-Bui AE, Schnapp BH, Smith LG (2021). Making our preference known: preference signaling in the emergency medicine residency application. West J Emerg Med.

[16] ACEP Now ACEP Surveys Members About COVID-19.

[17] Marco CA, Courtney DM, Ling LJ (2021). The emergency medicine physician workforce: projections for 2030. Ann Emerg Med.

[18] Katz B (2021). Survey of the Emergency Medicine Job Market. ACEP Now.

[19] Gettel CJ, Courtney DM, Janke AT (2022). The 2013 to 2019 emergency medicine workforce: clinician entry and attrition across the US geography. Ann Emerg Med.

[20] American Society of Anesthesiologists (1994). Estimation of Physician Workforce Requirements in Anesthesiology.

[21] Schubert A, Eckhout G, Cooperider T (2001). Evidence of a current and lasting national anesthesia personnel shortfall: scope and implications. Mayo Clin Proc.

[22] Reiter M, Allen BW (2020). The emergency medicine workforce: shortage resolving, future surplus expected. J Emerg Med.

[23] Haas MRC, Hopson LR, Zink BJ (2019). Too big too fast? Potential implications of the rapid increase in emergency medicine residency positions. AEM Educ Train.

