# Supplementary material for: Deciphering a Changing Match Environment in Emergency Medicine and Identifying Residency Program Needs
Source: West J Emerg Med. 2023 Jan 16;24(1):1–7. doi: 10.5811/westjem.2022.11.58060 (PMC9897243; doi:10.5811/westjem.2022.11.58060)
Supplement: Supplementary file 1 [file wjem-24-1-s001.docx]

**Appendix.** Survey instrument.

CORD Match 2022 Debriefing

Q1 CORD is trying to understand the needs of EM residency programs after the 2022 Match. Please fill out the following survey about your program’s match results, your needs and concerns for next year, and your interest in being involved in organizational initiatives. We recommend that program leadership (PD, APD, coordinator, VCE etc) complete this 5-10 minute survey.

In addition to informing organizational objectives, this survey has been given exempt status by the University of XXX IRB and the aggregate data may be used for research purposes.

Q2 What program do you represent? _________________________________

Q3 What is your leadership position in your program?

o PD (1)

o APD (2)

o CD/ACD (3)

o Assoc/Vice Chair for Education/Chair (4)

o Coordinator (5)

o Other (6) __________________________________________________

Q4 The format of my categorical EM program is:

o PGY 1-3 (1)

o PGY 1-4 (2)

Q5 How many positions did you put in the 2022 match for PGY-1’s?_______

Q30 The overall quality of our match was

o Substantially better than previous years (1)

o A little better (2)

o About the same (3)

o A little worse (4)

o Substantially worse than previous years (5)

Q6 Did your program fill in the 2022 match?

o Yes (1)

o No (2)

o Unsure (3)

Page Break

Skip To: Q17 If Did your program fill in the 2022 match? = Yes

Q7 How many initially unfilled positions did you have coming out of the main match?__________

Q8 How many of these unfilled positions did you fill in the SOAP?_____

Q9 How many of these unfilled positions did you fill OUTSIDE the SOAP?____

Q29 What factors do you feel were the biggest contributors to your program not filling this year? (Select up to 3 factors)

o Accreditation status of the program (1)

o Geographic location (2)

o Increasing numbers of EM programs (3)

o Interviewing/ranking insufficient numbers of candidates (5)

o New Leadership (6)

o New program (7)

o Program specific factors (e.g. wellness, curriculum changes etc) (8)

o Sponsoring institution/organization for the residency (e.g. university, private equity group, independent hospital) (9)

o Virtual interview format (10)

o Work force study/job prospects in EM (11)

o Other (12) __________________________________________________

Q10 How satisfied were you with the quality of candidates in the 2022 SOAP?

o Extremely satisfied (11)

o Somewhat satisfied (12)

o Neither satisfied nor dissatisfied (13)

o Somewhat dissatisfied (14)

o Extremely dissatisfied (15)

Q11 What were the biggest challenges you experienced with this year's SOAP?

________________________________________________________________

Page Break

Q17 Relative to previous years, the quality of our applicants was

o Substantially better (1)

o a little better (2)

o About the same (3)

o A little worse (4)

o Substantially worse (5)

Q12 Approximately how many applicants did you interview this season?

________________________________________________________________

Q13 Relative to 2021, the number of applicants interviewed was

o Substantially more (1)

o slightly more (2)

o The same (3)

o slightly less (4)

o substantially less (5)

Q14 Approximately, how many applicants did you place on your final ROL? ________

Q15 Relative to previous years, we went down our ROL in the match

o Substantially deeper (Higher number) (1)

o Slightly deeper (2)

o About the same (3)

o Slightly less (4)

o Substantially less deep (Lower number) (5)

Q16 Estimate how many ranks per position you went down your ROL if you filled. For example, a program with 10 slots who went to 100 on their ROL would report 10.0 (100/10). A program with 10 slots who went to 50 would be 5.0 (50/10) _________________________

Q18 As you plan for the 2022-23 Interview Season, how do you plan to change the number of interview positions for your program?

o Substantially Increase (1)

o Increase a little (2)

o Stay about the same (3)

o Decrease a little (4)

o Decrease substantially (5)

Q19 For your program, what is your goal number of interviews to conduct for your categorical EM program in 2022-23? ________________

Q28 As you reflect on the 2022 EM match, which factors do you think were the strongest contributors to the larger than usual number of unfilled slots in EM? Rank these factors in order with 1 being the highest contributor

______ Effects of COVID-19 pandemic (1)

______ Expansion of EM slots in existing programs (2)

______ Expansion of EM slots in new programs (3)

______ Perceptions of future job market (4)

______ Program-specific characteristics (5)

______ Virtual Interview Structure (6)

______ Other (7)

Page Break

Q21 What are 3 things that you would like to see CORD do to support programs in the changing match environment?

Q22 Idea #1

________________________________________________________________

Q23 Idea #2

________________________________________________________________

Q24 Idea #3

________________________________________________________________
